# Gastroesophageal Reflux Waning Over Time in Endoscopic Versus Surgical Myotomy for the Treatment of Achalasia: A Systematic Review and Meta-Analysis

**DOI:** 10.7759/cureus.31756

**Published:** 2022-11-21

**Authors:** Angelo So Taa Kum, Diogo Turiani Hourneaux De Moura, Igor Mendonça Proença, Masanori Aikawa, Sergio A Sánchez-Luna, Igor Braga Ribeiro, João Guilherme Ribeiro Jordão Sasso, Alexandre Moraes Bestetti, Wanderley Marques Bernardo, Eduardo G Hourneaux de Moura

**Affiliations:** 1 Serviço de Endoscopia Gastrointestinal do Departamento de Gastroenterologia, Hospital das Clínicas da Faculdade de Medicina da Universidade de São Paulo, São Paulo, BRA; 2 Center for Excellence in Vascular Biology, Brigham and Women’s Hospital, Harvard Medical School, Boston, USA; 3 Interventional, Metabolic & Surgical Endoscopy, University of Alabama at Birmingham Marnix E. Heersink School of Medicine, Birmingham, USA

**Keywords:** meta-analysis, systematic review, gastroesophageal reflux, heller myotomy, peroral endoscopic myotomy (poem), esophageal achalasia

## Abstract

Peroral endoscopic myotomy (POEM) and Heller myotomy with fundoplication (HMF) effectively treat achalasia, an esophageal motor disease. Although a significant number of meta-analyses have compared POEM and HMF, these studies showed discrepant postoperative gastroesophageal reflux disease (GERD) conclusions. This review aimed to objectively compare GERD over time, as well as the efficiency, safety, and adverse events in POEM versus HMF for treating achalasia.

We performed a systematic review and meta-analysis by searching Medline, Embase, Cochrane Library, Scopus, and Clinicaltrials.gov. The evaluated outcomes included early (within 12 months) and late (beyond 12 months) endoscopic assessment of GERD using the Lyon Consensus, clinical success, operative duration (OD), length of stay (LOS), and major adverse events (MAE).

A total of 29 observational studies and two randomized clinical trials (RCTs) with 13,914 patients were included. GERD was 28% higher among RCTs discussing POEM at early assessment (95%CI 0.02, 0.54) and was not different at late evaluation (95% confidence interval (CI) = 0.00, 0.22). No difference in reflux was observed among observational studies in both periods. The clinical success was 9% higher (95% CI = 0.05, 0.12), and the OD was 37.74 minutes shorter (95% CI = -55.44, -20.04) in POEM among observational studies, whereas it was not different among RCTs. The LOS and MAE were similar in the groups.

Comparisons among studies yielded divergent results. RCTs revealed that POEM had a higher incidence of GERD in the early assessment, whereas observational studies showed higher clinical success and a shorter OD in POEM. Ultimately, the between-group difference waned over time in GERD in all comparisons, resulting in no difference among RCTs in the late evaluation. Our meta-analysis demonstrated a non-preferential treatment of achalasia between endoscopic or surgical cardiomyotomy, prioritizing an individualized approach in the long term.

## Introduction and background

Achalasia results from the progressive degeneration of ganglion cells in the myenteric plexus in the esophageal wall, resulting in dysphagia, regurgitation, and thoracic pain. The treatment goal of achalasia during myotomy is the reduction of the lower esophageal sphincter (LES) pressure by partial or full-thickness dissection of muscle fibers of the esophagus and cardia.

Ernst Heller first reported cardiomyotomy in 1914, and after some improvements in the surgical technique, Heller myotomy with fundoplication (HMF) became the standard treatment for achalasia. Haruhiro Inoue first described peroral endoscopic myotomy (POEM) as a new treatment modality for achalasia in 2010. This procedure consists of making a submucosal tunnel endoscopically from the middle esophagus to the proximal stomach, followed by partial or full-thickness myotomy. POEM combines the benefits of a less invasive endoscopic treatment with similar efficacy and durability as the surgical myotomy. One major criticism of the procedure is that it does not involve an anti-reflux method, which may lead to an increased rate of postoperative gastroesophageal reflux disease (GERD) compared to HMF.

Although a significant number of meta-analyses have compared POEM and HMF, there is no unanimity regarding postoperative GERD, with some studies favoring surgery [1,2], others reporting similar results [3,4], and third parties reporting no concrete conclusions due to insufficient data or inconsistent reporting [5]. None of the previous studies have compared the reflux rate through time, whereas a waning between-group difference is noticed compared to a later assessment [6]. By applying the Lyon Consensus [7], an update of the clinical diagnosis of GERD, we aimed to perform a systematic review and meta-analysis with objective criteria comparing the incidence of GERD over time, as well as the efficiency, safety, and adverse events in endoscopic versus surgical cardiomyotomy.

## Review

Methodology

Protocol and Registration

This systematic review and meta-analysis were performed in conformity with the recommendations from the Cochrane Handbook of Systematic Reviews of Interventions [8] and the Preferred Reporting Items for Systematic Reviews and Meta-Analyses (PRISMA) guidelines [9]. The study protocol was registered in the International Prospective Register of Systematic Reviews (PROSPERO) database (https://www.crd.york.ac.uk/prospero/) under the file number CRD42021259233. It was approved by the Ethics Committee of Hospital das Clínicas, Faculty of Medicine at the University of São Paulo.

Information Source and Literature Search

Three authors conducted a systematic review of the literature data independently with individualized searches of Medline, Embase, Cochrane Library, Scopus, and Clinicaltrials.gov from their inception through September 2022. The following medical subject heading (MeSH) terms were used in each database: “(Heller Myotomy OR Heller OR Myotomy OR Cardiomyotomy OR Poem OR Peroral OR Per-oral OR Endoscopic OR Endoscopy) AND (Esophageal Achalasia OR Achalasia OR Achalasias OR Cardiospasm OR Megaesophagus).”

Study Selection and Data Items

Articles were included according to type, prospective or retrospective studies with abstract and full-text availability, regardless of date or language of publication; population, patients with the diagnosis of achalasia, independent of subtype, etiology, age, or prior treatment attempt; and types of intervention, POEM versus HMF. The primary outcomes included early (within 12 months) and late (beyond 12 months) assessment of postoperative GERD utilizing the esophagogastroduodenoscopy (EGD) findings through the Lyon Consensus [7], defined by LA grades B, C, and D, long-segment Barrett’s mucosa, or peptic esophageal stricture as a diagnosis of GERD. The secondary outcomes included clinical success, determined by a postoperative Eckardt Symptom Score (ESS) ≤3 [6]; operative duration (OD); length of stay (LOS); and major adverse events (MAE) based on Clavien-Dindo classification grades II to V [10]. The following exclusion criteria were also applied: secondary esophageal motility disorders, non-comparative studies on POEM and HMF, animal studies, and studies with abstracts only.

Data Extraction

All search hits, abstracts, and full-text manuscripts were evaluated for eligibility by the same three reviewers, each with experience in data extraction for retrospective and prospective studies, using the predefined inclusion and exclusion criteria. Any reviewer discrepancies were resolved by consensus discussion and arbitrated by the working group lead (ASTK). If the same research group published more than one article, it was decided to include the most updated data or both studies if there were different populations and complementary results. The data were included in Microsoft Excel tables. Data from the studies had the first author, year of publication, study design, period analyzed, the sample size in each procedure, follow-up time, mean age, gender percentage, and outcomes.

Risk of Bias and Quality of Evidence

Internal validation and the risk of bias in observational studies were performed using the Cochrane Risk of Bias in Non-randomized Studies of Interventions (ROBINS-I) tool [11]. For randomized clinical trials (RCTs), the analysis was performed using the Cochrane Risk of Bias 2.0 tool (RoB-2) [12]. The quality of the evidence was assessed using the standards from the Grading of Recommendations Assessment, Development, and Evaluation (GRADE) for each outcome using the GRADEpro - Guideline Development Tool software [13].

Statistical Analysis

The software Review Manager (RevMan) version 5.4.1 was used to compare RCTs and observational studies separately to analyze the outcomes. The effect sizes for continuous variables were analyzed using the mean difference (MD) and standard deviation (SD) with a 95% confidence interval (CI). The risk difference (RD) with a 95% CI was used for categorical variables. The RD and MD were statistically significant at a p-value of ≤0.05. If a study provided medians and interquartile ranges, the means and SD were described based on the McGrath method [14]. Heterogeneity among studies was assessed using the I^2^ index introduced by the Higgins method [15]. High heterogeneity was defined when I^2^ > 50%, and a random-effect model was performed. On the other hand, when statistical heterogeneity was not significant, I^2^ ≤ 50%, a fixed-effect model was used.

Results

Study Selection and Characteristics of Included Studies

The initial search identified 14,540 articles, resulting in 33 systematic reviews, composed of 30 observational studies [16-45] (Table 1) and three RCTs [6,46,47] (Table 2).

**Table 1:** Characteristics of the included observational studies. POEM: peroral endoscopic myotomy; MHF: Heller myotomy with fundoplication; NI: not informed; GERD: gastroesophageal reflux disease; EGD: esophagogastroduodenoscopy; OD: operative duration; LOS: length of stay; MAE: major adverse events

Study	Study type	Period analyzed	Sample size (POEM/HMF)	Follow-up (months, POEM/HMF)	Mean age (years, POEM/HMF)	Sex (% Male, POEM/HMF)	Outcomes analyzed
Akimoto et al. 2022 [16]	Retrospective	1996–2019	14/11	11/72	58/51	50/36	GERD by EGD, OD, MAE
Attaar et al. 2021 [17]	Prospective	2010–2020	126/33	60/60	64/58	49/58	OD, LOS, MAE
Bhayani et al. 2014 [18]	Prospective	2007–2012	37/64	≥6/≥6	56/57	52/48	OD, LOS, MAE
Caldaro et al. 2015 [19]	Prospective	2009–2013	9/9	13/31	12/11	34/67	GERD by EGD, clinical success, OD, LOS, MAE
Chan et al. 2016 [20]	Prospective	2000–2014	33/23	6/60	48/38	37/48	OD, LOS, MAE
Costantini et al. 2020 [21]	Prospective	2014–2017	140/140	24/31	47/48	50/52	GERD by EGD, clinical success, MAE
Docimo et al. 2017 [22]	Retrospective	2006–2015	44/122	NI	54/51	61/52	LOS
Fumagalli et al. 2016 [23]	Retrospective	1996–2015	6/9	5/19	71/49	50/34	Clinical success, OD, LOS, MAE
Greenleaf et al. 2018 [24]	Retrospective	2003–2016	20/21	11/65	60/58	60/48	MAE
Hanna et al. 2018 [25]	Retrospective	2011–2016	42/54	22/37	51/53	64/37	GERD by EGD, clinical success
Hungness et al. 2013 [26]	Prospective	2004–2012	18/55	6/6	38/49	72/53	OD, LOS, MAE
Kahaleh et al. 2020 [27]	Prospective	2014–2019	69/64	12/12	47/46	42/47	Clinical success, MAE
Khashab et al. 2017 [28]	Retrospective	2009–2014	52/52	16/9	47/47	52/54	Clinical success, MAE
Khoraki et al. 2022 [29]	Retrospective	2015–2018	1,715/9,555	NI	55/56	48/49	LOS, MAE
Kumagai et al. 2015 [30]	Prospective	2012–2013	42/41	12/≥6	46/45	64/54	OD, LOS, MAE
Kumbhari et al. 2015 [31]	Retrospective	2000–2013	49/26	9/22	58/52	59/50	Clinical success, OD, LOS, MAE
Leeds et al. 2017 [32]	Prospective	2014–2017	12/11	12/10	52/53	33/54	Clinical success, OD, LOS, MAE
Miller et al. 2017 [33]	Retrospective	2011–2015	98/27	NI	NI	NI	LOS
de Pascale et al. 2017 [34]	Retrospective	2012–2015	32/42	24/27	56/48	37/55	GERD by EGD, clinical success, OD, LOS, MAE
Peng et al. 2017 [35]	Retrospective	2009–2012	13/18	46/54	38/45	62/44	Clinical success, OD, LOS, MAE
Podboy et al. 2021 [36]	Retrospective	2010–2015	55/43	48/64	59/58	40/23	GERD by EGD, clinical success, LOS, MAE
Ramirez et al. 2018 [37]	Prospective	2010–2016	50/55	10/20	50/45	30/36	GERD by EGD, clinical success, MAE
Schneider et al. 2016 [38]	Retrospective	2004–2016	25/25	8/36	50/54	52/48	GERD by EGD, clinical success, OD, MAE
Shea et al. 2020 [39]	Retrospective	2009–2018	44/97	18/45	52/52	60/60	Clinical success, OD
Teitelbaum et al. 2015 [40]	Prospective	2013	36/20	11/12	50/53	69/45	Clinical success
Trieu et al. 2021 [41]	Retrospective	2017	580/2,850	NI	56/54	53/48	LOS, MAE
Ujiki et al. 2013 [42]	Prospective	2009–2013	18/21	4/5	64/60	72/57	Clinical success, OD, LOS, MAE
Ward et al. 2017 [43]	Prospective	2011–2015	41/24	≥12/≥12	63/62	61/58	Clinical success, LOS
Ward et al. 2021 [44]	Prospective	2015–2019	54/46	10/10	57/54	35/28	GERD by EGD, clinical success
Wirsching et al. 2019 [45]	Prospective	2014–2017	23/28	6/6	58/57	48/43	Clinical success, OD, LOS, MAE

**Table 2:** Characteristics of the included randomized controlled trials. POEM: peroral endoscopic myotomy; HMF: Heller myotomy with fundoplication; GERD: gastroesophageal reflux disease; EGD: esophagogastroduodenoscopy; OD: operative duration; LOS: length of stay; MAE: major adverse events

Study	Period analyzed	Sample size (POEM/HMF)	Follow-up (months, POEM/HMF)	Mean age (years, POEM/HMF)	Sex (% Male, POEM/HMF)	Outcomes analyzed
Conte et al. 2020 [46]	2017–2018	20/20	≥12/≥12	45/44	40/30	GERD by EGD, clinical success, OD, LOS, MAE
de Moura et al. 2022 [47]	2017–2018	20/20	≥12/≥12	45/44	40/30	GERD by EGD, clinical success; OD, LOS, MAE
Werner et al. 2019 [6]	2012–2015	112/109	≥24/≥24	49/49	61/55	GERD by EGD, clinical success, OD, LOS, MAE

**Figure 1:**
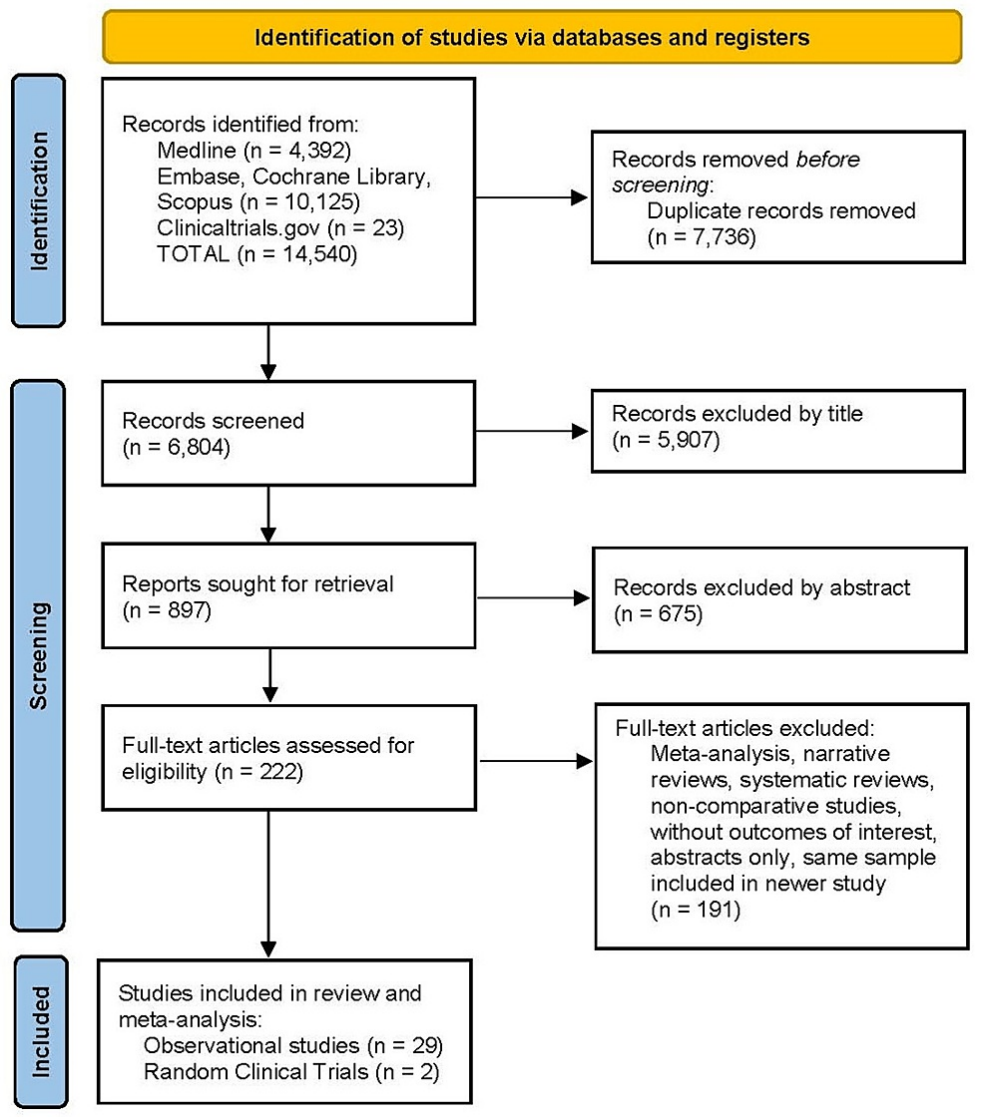
PRISMA flow diagram showing the study selection process. PRISMA: Preferred Reporting Items for Systematic Reviews and Meta-Analyses

The study by Trieu et al. [41] was excluded from the meta-analysis by evaluating the same database already included in Khoraki et al. [29]. The same occurred for the study by Conte et al. [46], which was replaced by de Moura et al. [47], who published updated data from the same sample. The final PRISMA flow diagram (Figure 1) resulted in 29 observational studies [16-40,42-45] and two RCTs [6,47], evaluating 3,049 patients who underwent POEM and 10,865 who underwent HMF, totaling 13,914 patients.

Risk of Bias and Quality of Evidence

**Figure 2:**
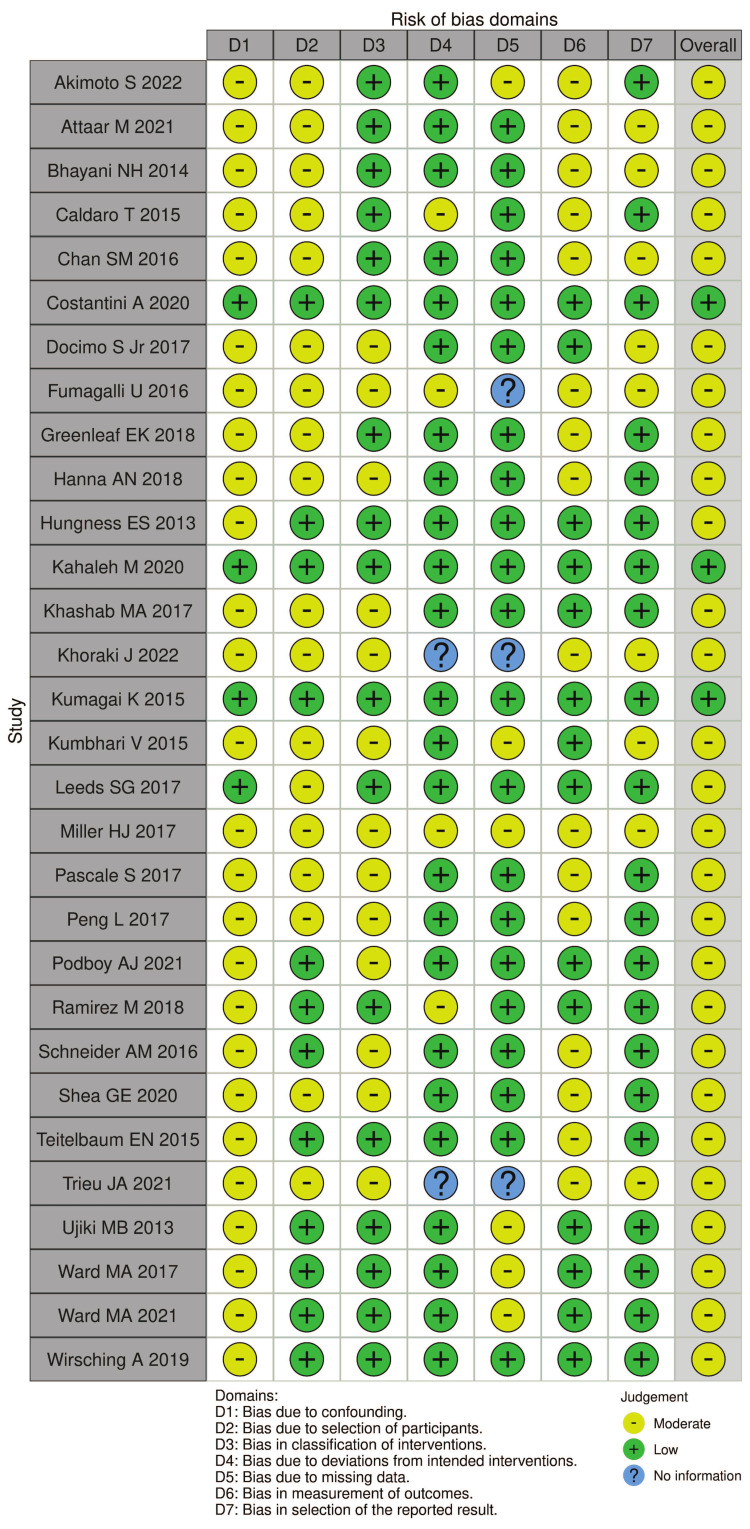
Cochrane Risk of Bias in Non-randomized Studies of Interventions. Studies represented in the figure [16-45].

The risk of bias between observational studies was moderate, except for Costantini et al. [21], Kahaleh et al. [27], and Kumagai et al. [30], whose overall risk of bias was low (Figure 2).

**Figure 3:**
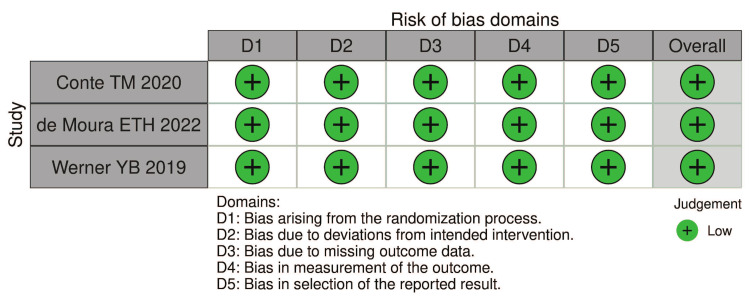
Cochrane Risk of Bias Tool 2.0. Studies represented in the figure [6,46,47].

The RCTs data presented a low risk of bias (Figure 3).

The quality of evidence assessed by GRADE resulted in each outcome and comparison among observational studies and RCTs (Table 3).

**Table 3:** Quality of evidence assessed by GRADE. The risk in the intervention group (and its 95% CI) is based on the assumed risk in the comparison group and the relative effect of the intervention (and its 95% CI). CI: confidence interval; MD: mean difference; RCTs: randomized controlled trials; LA: Los Angeles classification; GERD: gastroesophageal reflux disease; GRADE: Grading of Recommendations Assessment, Development, and Evaluation a: heterogeneity >50% and ≤75%; b: the CI crosses the clinical decision threshold between recommending and not recommending intervention; c: heterogeneity >75%

Outcomes	Number of participants (studies) follow-up	Certainty of the evidence (GRADE)	Anticipated absolute effects	
			Risk with surgical myotomy (Heller)	Risk difference with endoscopic myotomy (POEM)
Early endoscopic findings of GERD (within 12 months) - observational studies	316 (5 observational studies)	⨁⨁⨁◯ Moderate^a,b^	69 per 1.000	69 fewer per 1.000 (69 fewer to 69 fewer)
Early endoscopic findings of GERD (within 12 months) - randomized controlled trials	255 (2 RCTs)	⨁⨁⨁⨁ High^a^	47 per 1.000	47 fewer per 1.000 (47 fewer to 47 fewer)
Late endoscopic findings of GERD (beyond 12 months) - observational studies	138 (2 observational studies)	⨁◯◯◯ Very low^b,c^	63 per 1.000	63 fewer per 1.000 (63 fewer to 63 fewer)
Late endoscopic findings of GERD (beyond 12 months) - randomized controlled trials	256 (2 RCTs)	⨁⨁⨁⨁ High	94 per 1.000	94 fewer per 1.000 (94 fewer to 94 fewer)
Clinical success (Eckardt symptom score of 3 or less) - observational studies	1,343 (19 observational studies)	⨁⨁⨁⨁ High	808 per 1.000	808 fewer per 1.000 (808 fewer to 808 fewer)
Clinical success (Eckardt symptom score of 3 or less) - randomized controlled trials	261 (2 RCTs)	⨁⨁⨁◯ Moderate^b^	860 per 1.000	860 fewer per 1.000 (860 fewer to 860 fewer)
Operative duration (minutes) - observational studies	1,014 (16 observational studies)	⨁⨁◯◯ Low^c^	The mean operative duration (minutes) - observational studies was 0	MD 37.74 lower (55.44 lower to 20.04 lower)
Operative duration (minutes) - randomized controlled trials	261 (2 RCTs)	⨁◯◯◯ Very low^b,c^	The mean operative duration (minutes) - randomized controlled trials was 0	MD 67.31 lower (175 lower to 40.38 higher)
Length of hospital stay (days) - observational studies	12,522 (18 observational studies)	⨁◯◯◯ Very low^b,c^	The mean length of hospital stay (days) - observational studies was 0	MD 0.34 lower (0.83 lower to 0.14 higher)
Length of hospital stay (days) - randomized controlled trials	261 (2 RCTs)	⨁⨁⨁◯ Moderate^b^	The mean length of hospital stay (days) - randomized controlled trials was 0	MD 0.31 lower (0.67 lower to 0.05 higher)
Major adverse events (Clavien-Dindo grades II to V) - observational studies	12,904 (22 observational studies)	⨁⨁⨁◯ Moderate^a,b^	81 per 1.000	81 fewer per 1.000 (81 fewer to 81 fewer)
Major adverse events (Clavien-Dindo grades II to V) - randomized controlled trials	261 (2 RCTs)	⨁⨁◯◯ Low^a,b^	70 per 1.000	70 fewer per 1.000 (70 fewer to 70 fewer)

Meta-analysis

Primary Outcomes

**Figure 4:**
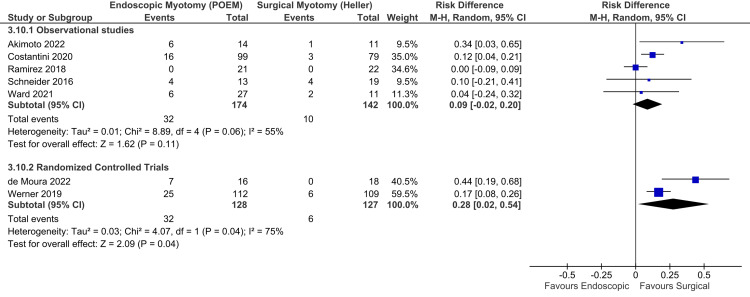
Early endoscopic findings of GERD (within 12 months). Studies represented in the forest plot: five observational studies [16,21,37,38,44] and two randomized controlled trials [6,47]. POEM: peroral endoscopic myotomy; M-H: Mantel-Haenszel; CI: confidence interval; GERD: gastroesophageal reflux disease

Postoperative GERD - endoscopic findings based on the Lyon Consensus: In the early endoscopic findings, adequate data were found in seven studies, consisting of five observational studies [16,21,37,38,44] and two RCTs [6,47], totaling 571 patients. There was high heterogeneity among the studies. Thus, the random-effect model was used (Figure 4). Among the observational studies, there was no difference in GERD between the groups (RD = 0.09; 95% CI = -0.02, 0.20; I^2^ = 55%; p = 0.11), supported by a moderate quality of evidence. Among the RCTs, there was a higher GERD in POEM (RD = 0.28; 95% CI = 0.02, 0.54; I^2^ = 75%; p = 0.04), supported by a high quality of evidence.

**Figure 5:**
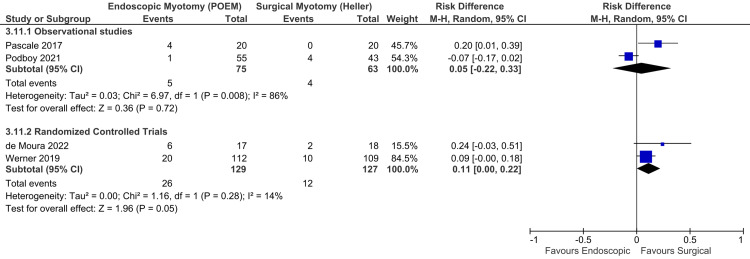
Late endoscopic findings of GERD (beyond 12 months) Studies represented in the forest plot: two observational studies [34,36] and two randomized controlled trials [6,47] POEM: peroral endoscopic myotomy; M-H: Mantel-Haenszel; CI: confidence interval; GERD: gastroesophageal reflux disease

In the late endoscopic findings, adequate data were found in four studies, consisting of two observational studies [34,36] and two RCTs [6,47], totaling 394 patients. There was high heterogeneity among the studies. Thus, the random-effect model was used (Figure 5). Among observational studies, there was no difference in GERD between the groups (RD = 0.05; 95% CI = -0.22, 0.33; I^2^ = 86%; p = 0.72), supported by a very low quality of evidence. Among RCTs, there was no difference between the groups (RD = 0.11; 95% CI = 0.00, 0.22; I^2^ = 14%; p = 0.05), supported by a high quality of evidence.

Secondary Outcomes

**Figure 6:**
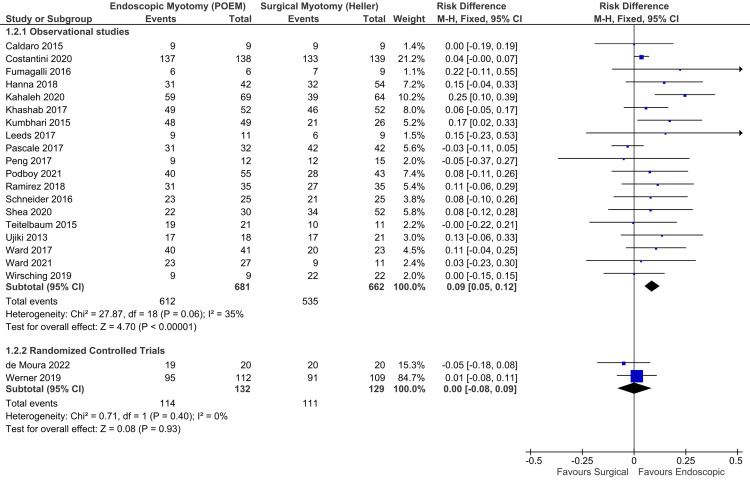
Clinical success (Eckardt symptom score ≤3). Studies represented in the forest plot: 19 observational studies [19,21,23,25,27,28,31,32,34-40,42-45] and two randomized controlled trials [6,47]. POEM: peroral endoscopic myotomy; M-H: Mantel-Haenszel; CI: confidence interval

Clinical success: Adequate data were found in 21 studies, consisting of 19 observational studies [19,21,23,25,27,28,31,32,34-40,42-45] and two RCTs [6,47], totaling 1,604 patients. There was no significant heterogeneity. Thus, the fixed-effect model was used (Figure 6). Among observational studies, the clinical success was higher in endoscopic myotomy (RD = 0.09; 95% CI = 0.05, 0.12; I^2^ = 35%; p < 0.01), with a number needed to treat (NNT) of 11.1, supported by a high quality of evidence. Among RCTs, there was no difference between POEM and HMF (RD = 0.00; 95% CI = -0.08, 0.09; I^2^ = 0%; p = 0.93), supported by a moderate quality of evidence.

**Figure 7:**
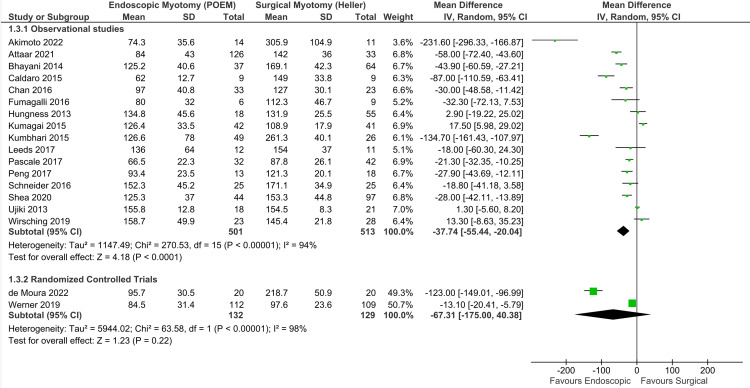
Operative duration (minutes). Studies represented in the forest plot: 16 observational studies [16-20,23,26,30-32,34,35,38,39,42,45] and two randomized controlled trials [6,47]. POEM: peroral endoscopic myotomy; M-H: Mantel-Haenszel; CI: confidence interval

Operative duration: A total of 18 studies analyzed OD, consisting of 16 observational studies [16-20,23,26,30-32,34,35,38,39,42,45] and two RCTs [6,47], with a total of 1,275 patients. There was high heterogeneity among the studies. Thus, the random-effect model was used (Figure 7). In observational data, POEM had a shorter OD than HMF (MD = -37.74 minutes; 95% CI = -55.44, -20.04; I^2^ = 94%; p < 0.01), supported by a low quality of evidence. Among RCTs, there was no difference in the groups (MD = -67.31 minutes; 95% CI = -175.00, 40.38; I^2^ = 98%; p = 0.22), supported by a very low quality of evidence.

**Figure 8:**
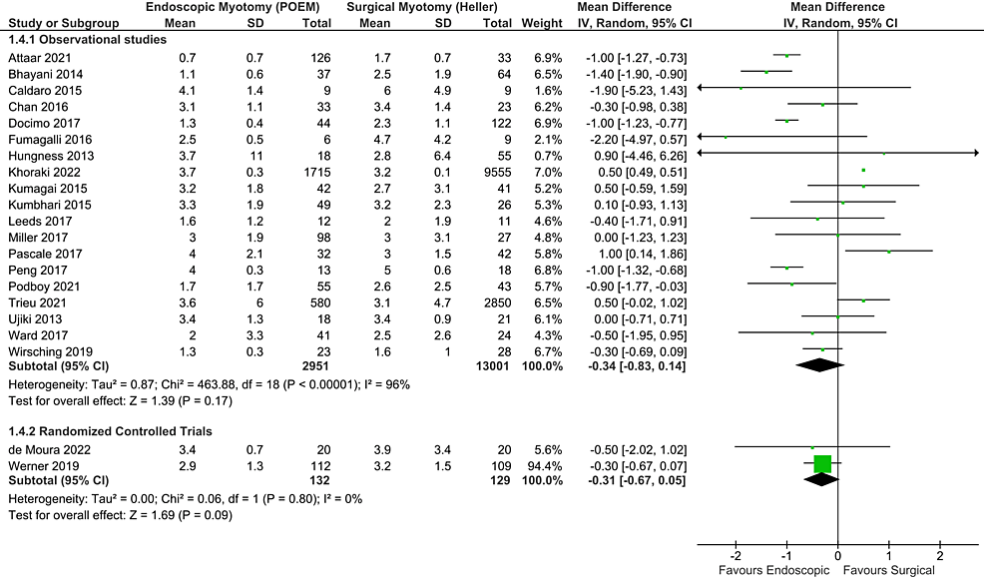
Length of stay (days). Studies represented in the forest plot: 18 observational studies [17-20,22,23,26,29-36,42,43,45] and two randomized controlled trials [6,47]. POEM: peroral endoscopic myotomy; M-H: Mantel-Haenszel; CI: confidence interval

Length of stay: Adequate data were available in 20 studies, consisting of 18 observational studies [17-20,22,23,26,29-36,42,43,45] and two RCTs [6,47], totaling 12,783 patients. There was significant heterogeneity among the studies. Thus, a random-effect model was used (Figure 8). Among observational studies, there was no difference in LOS between the groups (MD = -0.40 day; 95% CI = -0.91, 0.11; I^2^ = 96%; p = 0.12), supported by a very low quality of evidence. Among RCTs, there was no difference between the groups (MD = -0.31 day; 95% CI = -0.67, 0.05; I^2^ = 0%; p = 0.09), supported by a moderate quality of evidence.

**Figure 9:**
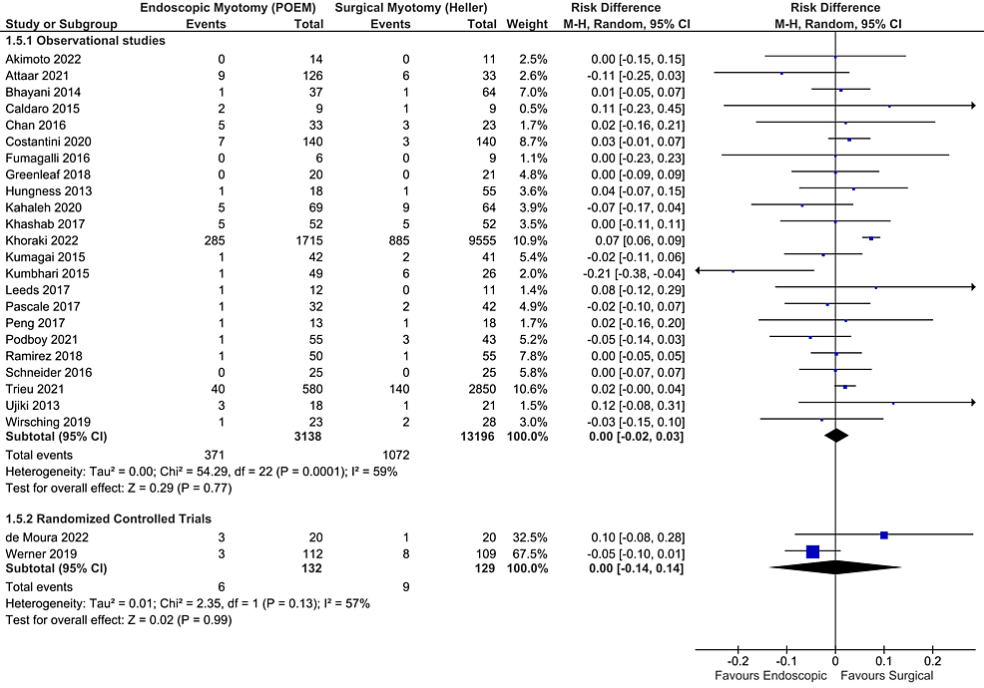
Major adverse events (Clavien-Dindo grades II to V). Studies represented in the forest plot: 22 observational studies [16-21,23,24,26-32,34-38,42,45] and two randomized controlled trials [6,47]. POEM: peroral endoscopic myotomy; M-H: Mantel-Haenszel; CI: confidence interval

Major adverse events: Adequate data were available in 24 studies, consisting of 22 observational studies [16-21,23,24,26-32,34-38,42,45] and two RCTs [6,47], totaling 13,165 patients. Because there was high heterogeneity among the studies, the random-effect model was used (Figure 9). Among observational studies, there was no difference between both approaches in the occurrence of major complications (RD = 0.00; 95% CI = -0.03, 0.03; I^2^ = 60%; p = 0.99), supported by a moderate quality of evidence. Among RCTs, there was also no difference between both groups (RD = 0.00; 95% CI = -0.14, 0.14; I^2^ = 57%; p = 0.99), supported by a low quality of evidence.

Discussion

This is the first systematic review with a meta-analysis to evaluate GERD over time by comparing endoscopic versus surgical myotomy for treating achalasia based on the Lyon Consensus recommendations [7]. Our study demonstrated dichotomous results. POEM and HMF did not have GERD rates among observational studies, while RCTs showed a lower incidence in the surgical group in the early assessment, which reduced to no difference in the late evaluation. The clinical success was higher, and the OD was lower in the endoscopic group among observational studies, while RCTs revealed no difference in these outcomes. Nonetheless, the LOS and MAE showed no difference in all comparisons.

Postoperative GERD is a primary concern involving cardiomyotomy. However, available meta-analyses [1-5] report heterogeneous results. The anamnesis, questionnaire data on quality of life, and response to antisecretory therapy are all resumed as subjective data, insufficient to make a conclusive diagnosis of GERD in isolation. With the available articles to analyze, the acid exposure time in the pHmetry was evaluated with the outdated threshold of 4%, not the 6% defined by the Lyon Consensus [7]. Furthermore, an important confounding factor is that the megaesophagus morphology developed over time maintains partial food retention in the esophagus, leading to the acid fermentation process, which lowers the intraluminal pH and alters the pHmetry examination even after the cardiomyotomy. The only way to use this method as a solid criterion for GERD is to review a 24-hour pHmetry manually. In contrast, an automated review overestimated acid exposure time by not differentiating actual reflux from fermentation [48]. Therefore, we decided to discard this examination for further analysis.

EGD was the prime evidence used in our study to analyze GERD objectively using the Lyon Consensus [7]. The time frame of 12 months was the available period to divide studies between early and late assessments and conduct a feasible comparison. Therefore, further analysis of other periods may be possible when more reliable studies are published. Our results confirmed the decreasing difference in GERD between groups over time [49], a relevant event noticed among observational and randomized studies, even nullifying the disparity between POEM and HMF in the late assessment among RCTs. Although the use of proton-pump inhibitors (PPIs) is higher in the endoscopic approach [6], their use is not the only explanation, as the treatment period of GERD is well established within two months, and the reduction difference between groups of reflux is noted beyond the one-year follow-up. Because achalasia generates the opposite symptom of reflux by acting as a natural barrier, cardiomyotomy could imply acid exposure to a more sensitive esophageal mucosa, which was already affected by food stasis fermentation, with microscopic changes mimicking reflux and lymphocytic esophagitis [50]. A hypothesis to this waning between-group difference over time might be attributed to a late esophageal remodeling, which involves the improvement of the esophageal wall tortuosity, the lumen diameter reduction [51], and improvement in the clearance of food stasis, which prevents fermentation and decreases GERD in the endoscopic group. Another explanation could be the wrap loosening in the HMF group over time, which may lead to intrathoracic migration or hiatal hernia recurrence, partial or complete wrap disruption, and fundoplication failure [52].

The ESS ≤3 is widely considered a treatment success [53] and is selected to assess the efficacy of cardiomyotomy. This outcome favored POEM over HMF among observational studies, emphasizing some of the proponents of the endoscopic approach, who argue that preserving the diaphragmatic hiatus as a natural barrier prevents GERD, in contrast to the surgical procedure, which violates the hiatus to extend the myotomy along the esophagus [44,45]. Moreover, POEM has an advantage within specific achalasia phenotypes, especially type III achalasia and other spastic disorders of the esophagus, due to its ability to create a longer or tailored myotomy to the spastic segment, as determined by high-resolution myotomy with easier access in the proximal esophagus when compared to the surgical approach [44]. The absence of comparison between achalasia types in the studies made this analysis unfeasible. The higher clinical success in the endoscopic group could be correlated with a higher prevalence of type III achalasia in observational studies, with one study [31] comparing this type exclusively. Nonetheless, the non-difference between groups among RCTs might be attributed to the restricted number of two studies.

The OD and LOS had the highest heterogeneity among the outcomes, primarily associated with the variable expertise among physicians and multidisciplinary teams, the internal protocols, and the postoperative support of each institution. The disparity between studies regarding OD may be related to the fact that POEM is a newer technique than HMF and, therefore, is directly correlated with the expertise of the endoscopist involved. Despite no difference between groups in the LOS, the shorter OD in the POEM noticed only among observational studies might be associated with the restricted number of RCTs.

Regarding safety profile, common intraoperative complications solved at the procedure, such as esophageal or gastric perforation and limited bleeding, all treated intraprocedurally, were counted as MAE whenever there was a need for longer LOS or additional treatment, based on the Clavien-Dindo classification of surgical complications [10]. Similar to previous meta-analyses [3,5], the MAE was identical between groups in all comparisons.

Despite following strict methodological guidelines, our study has some limitations. The main restriction is related to the lack of high-quality data available in the literature, including a limited number of RCTs. Other limitations were the absence of manually reviewed 24-hour pHmetry in the general studies for comparison; the EGD evaluation being available in a limited number of articles; the non-specified use of PPIs in postoperative patients; the lack of isolated comparison between achalasia types; and not considering the diagnosis of GERD in mild esophagitis (LA grade A) associated with pHmetry or subjective reflux symptoms (e.g., heartburn and acid regurgitation), which mimic other achalasia symptoms.

Our study showed a decreasing GERD difference over time among endoscopic and surgical myotomy, which highlights the benefits of immediate post-POEM PPI use with the possibility of gradual discontinuation during the follow-up. This result may broaden the endoscopic indication to treat achalasia, primarily type III, if the patient is not concerned about using PPI after the procedure.

## Conclusions

Comparisons among randomized and observational studies yielded divergent results. Based on the higher statistical significance and quality of evidence, RCTs revealed that POEM had a higher incidence of GERD in the early assessment. In contrast, observational studies showed higher clinical success and a shorter OD in the endoscopic approach. The LOS and MAE were similar in the groups. Ultimately, the between-group difference waned over time in GERD in all comparisons, resulting in no difference among RCTs in the late evaluation. Our meta-analysis demonstrated a non-preferential treatment of achalasia between endoscopic or surgical cardiomyotomy, prioritizing an individualized approach in the long term.
